# Review of the Roles and Interaction of Androgen and Inflammation in Benign Prostatic Hyperplasia

**DOI:** 10.1155/2020/7958316

**Published:** 2020-10-28

**Authors:** Yu Tong, Ren-yuan Zhou

**Affiliations:** Department of Urology, Jing'an District Central Hospital, Fudan University, Shanghai 200040, China

## Abstract

The lower urinary tract symptoms (LUTSs) and acute urinary retention (AUR) caused by benign prostatic hyperplasia (BPH) can seriously affect the quality of life of elderly men. Studies suggest that both androgens and inflammation greatly influence the occurrence and development of BPH in most patients. These two factors combined can also affect each other, leading to pathological changes in the stromal and epithelial tissue of the prostate transition zone in BPH patients. DHT in the prostate tissue of BPH patients may activate a chronic inflammatory response in the prostate, amplifying the expression of inflammatory factors and upregulating the proliferation ability of prostate tissue.

## 1. Introduction

Benign prostatic hyperplasia (BPH) is the most common urinary system disease in elderly men [1], and its main pathological manifestations are proliferative hypertrophy and nodule formation in the transition zone of the prostate. According to American epidemiological studies, the number of patients diagnosed with BPH reached 30,555,010 cases between 2004 and 2013 [2]. The occurrence and development of BPH are closely related to age. Autopsy pathological statistics have shown that the proportion of 51–60-year-old men with BPH is about 42%, and the proportion in men over 80 years old rises to about 85% [3]. BPH is the most common cause of lower urinary tract symptoms (LUTSs) in men. LUTSs include the symptoms of urination and storage, which seriously affect quality of life [4]. At the same time, BPH also increases the risk of urinary tract infection (UTI) and acute urinary retention (AUR). Although the cellular and molecular mechanisms of BPH are still unclear, changes in androgen levels and senility-related tissue remodeling have been considered to be important factors contributing to homeostasis disorders in the prostate [5]. In addition, some evidence suggests that metabolic syndrome and chronic inflammation also play a role in the occurrence and progression of BPH/LUTS [6].

This review focuses on the interactions between androgens and inflammation as well as their individual roles in BPH and LUTS development and explores the relationship between androgens and inflammation in BPH pathogenesis.

## 2. The Role of Androgens in BPH

When the hypothalamic pituitary gonad axis activates in puberty, the testes begin to synthesize large amounts of testosterone (T), and the prostate gland develops rapidly, increasing in both volume and weight [7]. After entering adulthood, prostate grows plateaus and slows down, but after the age of about 40, the volume of the prostate may begin to increase gradually in some men. Many studies have confirmed that androgens can directly affect prostate tissues and participate in BPH development. Sasagawa and his research team observed 13 patients with hypogonadism (aged 25–32) continuously. Before treatment, their prostate volumes were significantly smaller than those of healthy men of the same age [8]. After exogenous testosterone supplementation, a significant increase in prostate volume was observed in the patients with hypogonadism [8, 9]. Pejcic et al. [10] performed prostate biopsies on 93 patients with BPH, and liquid chromatography mass spectrometry (LC-MS) was used to detect the androgen concentration in prostate tissue. T and dihydrotestosterone (DHT) levels were both found to be significantly higher in larger prostates (total prostate volume [TPV] > 30mL) than in smaller ones (TPV < 30mL) (T: 1.05 ± 0.75 and 0.46 ± 0.29 ng/g; DHT: 15.0 ± 6.09 and 4.51 ± 2.75ng/g). There were significant correlations between the levels of T and TPV (*r* = 0.71) and between the levels of DHT and TPV (*r* = 0.74) [10].

In androgen-dependent tissues, such as brain, skeletal muscle, and seminiferous epithelium, androgens mainly participate in androgen-dependent processes in the form of testosterone [11]. In prostate tissue, however, testosterone is catalyzed by 5*α*-reductase (5*α*-R), which is synthesized by prostate stromal cells, to convert to more potent DHT, which plays a key role in organ development and function [7]. The studies also found that, whether in healthy adults or elderly BPH patients, the level of DHT in prostate tissue is about 10–20 times higher than the T level, while the opposite is true in serum [10, 12]. In the PLESS study, 1,524 BPH patients were given 5 mg finasteride (a 5*α*-reductase inhibitor) daily, and 1,516 BPH patients received a placebo. Four years later, prostate volume had decreased by 18% in the finasteride group, but increased by 14% in the placebo group [13]. Roehrborn et al. [14] also observed similar results. They randomly distributed 4,325 BPH patients into a dutasteride group and a control group. After two years of treatment, it was found that the serum DHT levels had decreased by 90.2% in patients taking dutasteride, but serum DHT levels increased by 9.6% in the placebo group. The volume of the prostate transition zone decreased by 25.7% and increased by 12.4% in the dutasteride and control groups, respectively. A similar phenomenon was also observed in experimental animals. Berry et al. [15] found that the prostate volume of castrated dogs increased after exogenous androgen supplementation. Prostate volume shrunk after older dogs with BPH were castrated, but increased again after exogenous androgen supplementation. After injecting testosterone subcutaneously into mice and constructing a BPH mouse model, it was observed that the prostates of mice were enlarged significantly, the morphological structure of prostate cells changed, and the stromal cells and epithelial cells became hypertrophic. The expression of antiapoptotic gene Bcl-2 in the prostates of mice in the BPH group was significantly upregulated, while the expression of apoptosis-related genes, such as Bax, p53, and caspase-3, were downregulated, resulting in cellular proliferation and BPH occurrence [16, 17]. Leimgruber et al. [18] proved *in vitro* that the proliferation capacity of rat prostate smooth muscle cells (pSMCs) was significantly enhanced, and the expression of proliferation-related protein p-ERK1/2 increased after testosterone treatment. The above results indicate that androgens are involved in the proliferation of prostate tissue. Reducing androgen levels can inhibit further prostate enlargement, and thus, reduce prostate volume.

As age increases, the serum androgen level of elderly men gradually decreases, while BPH incidence gradually increases. This phenomenon appears contradictory, but is actually related to the fact that the DHT level in the prostate is not affected by age. When androgen levels in the serum and prostate of BPH patients were measured separately, the results showed that there was no significant correlation between the level of serum T and the DHT level in prostate tissue [19]. Cook and his colleagues [20] analyzed androgen levels in the serum and prostate tissue of 251 patients with prostate cancer, and they found that serum androgen concentration could not predict the level of androgens in prostate tissue well. More interestingly, Thirumalai et al. [12] used drugs to suppress androgens in 51 healthy men (aged 22–55) and then supplemented them with exogenous T at different concentrations (1.25g, 2.5g, 5.0g, 10g, or 15g daily) for 12 weeks. Exogenous T resulted in a dose-dependent increase in serum T and DHT concentrations. The higher the supplemental exogenous testosterone concentration, the more significant the increase in serum T level. As for intraprostatic androgens, regardless of supplemental T dose, intraprostatic DHT level was comparable and was 10 to 20 times higher than intraprostatic T. There was no obvious correlation between the level of intraprostatic DHT and the level of supplemented exogenous T; the intraprostatic DHT was always maintained at a relatively stable level. This evidence indicates that DHT can be efficiently converted from T and retained stably in the prostate tissue, even if the serum T level fluctuates in a broad physiological range. This finding may explain why the prostate continues to be stimulated by DHT after peripheral blood T declines in elderly men. The concentration of DHT in prostate tissue can more accurately reflect the effect of androgens on tissue proliferation.

DHT alone is not functional; a combination with androgen receptor (AR) is required to achieve the corresponding biological effects. The level of DHT in the prostate tissue of BPH patients may not decrease with age, but AR expression changes. Nicholson et al. [21] observed 52 specimens from BPH patients after prostatectomy and found that the expression of AR was significantly higher than the AR expression of normal prostate tissue. The number of AR-positive cells and the density of AR staining in epithelial cells and stromal cells both increased. Monti et al. [22] further observed that both androgen concentration and AR expression levels were highest in the periurethral prostate (the main site of hyperplasia). This indicates that androgens may be more likely to bind to the receptor and activate downstream reaction pathways in BPH tissues, especially around the urethra, resulting in corresponding biological effects. Recently, researchers have found that there are two different AR subtypes in smooth muscle cells. The AR subtype in the cytoplasm is involved in the regulation of proliferation and inflammation, while another AR subtype on the cell membrane has a stronger promoting effect on cell proliferation [23]. As age increases, changes in the homeostasis of the prostate may cause changes in the expression levels of different AR subtypes. These changes may increase an androgen's promotion of prostate epithelial and stromal cell proliferation, resulting in further BPH progression.

The above results indicate that androgens, represented by DHT in prostate tissue, play an important role in the pathological progression of BPH. However, a decrease in serum androgen and serum T levels does not effectively change the level of androgens (especially DHT) in prostate tissue. An increase in AR expression and changes in expressions of AR subtype in BPH tissues can also strengthen the intraprostatic DHT-promoting effect, leading to the proliferation of prostate tissues.

## 3. The Role of Inflammation in BPH

After middle age, the total volume of the prostate increases with age [24]. Clinical practice has proven that taking 5*α*-reductase inhibitor (5AR-I) can effectively reduce the level of DHT in prostate tissue and reduce the risk of BPH progression, but 10% of patients (17% of the control group) still have clinical progress [14, 25]. This shows that androgens are not the only factors that cause BPH/LUTS. To accurately select an effective treatment for patients, it is necessary to distinguish the roles of androgens and other factors in BPH and to discover the mechanism behind the other factors. Through observing prostate tissue biopsy specimens and surgical specimens of patients with prostate disease, chronic inflammation was found in BPH tissue. Zlotta et al. [26] anatomized the cadavers of 100 Asian males and 320 Caucasian males and observed chronic inflammation in the prostate in more than 70% of autopsy specimens in both groups. Multivariate statistical analysis showed that those with chronic inflammation in the prostate had a seven-fold higher risk of BPH than those without inflammation (HR: 6.84; 95% CI: 4.05–11.78; *P* < 0.0001) [26]. Experimental data from MTOPS showed that chronic inflammation infiltration was found in about 40% of BPH biopsy specimens, and these people had higher serum PSA levels and larger prostate volumes [25, 27]. The REDUCE trial reported similar findings. Prostate biopsies were performed on 8,824 patients, and chronic inflammation was observed in 77.6% of the samples [28, 29]. Thus, inflammation was widespread in the prostates of BPH patients, and the degree of inflammation was also positively correlated with prostate volume.

According to clinical observations, chronic inflammation in the prostate is a common risk factor for benign prostate enlargement (BPE) and LUTS, in addition to intraprostatic DHT. Analysis of the REDUCE trial, which utilized a large sample, showed that prostate inflammation was positively correlated with prostate volume (46.5mL vs. 43.4mL; *P* < 0.0001) and international prostate symptom score (IPSS) (8.8 vs. 8.2; *P* < 0.0001) [28, 29]. Robert et al. [30] observed specimens from 282 patients undergoing BPH surgery and found significant correlation between the degree of prostate inflammation, prostate volume, and IPSS. The mean prostate volume in the low-inflammation group was 62 mL, while that of the high-inflammation group was 77mL (*P* = 0.002). Also, the average IPSSs of the low-inflammation and high-inflammation groups were 12 and 21 (*P* = 0.02), respectively. In addition to grade of prostatic inflammation, recent studies have found that differences in the distribution of inflammation in the prostate's transition zone may also influence the severity of clinical symptoms in patients. By observing the TURP and HoLEP samples of 179 BPH patients, the patients were divided into stromal group and nonstromal groups according to whether or not the main site of chronic inflammation occurred in the stromal tissue. The stromal group was found to have a significantly larger total prostate volume (TPV) than the nonstromal group (63.8 vs. 53.8mL; *P* = 0.032), and the incidence of AUR was significantly increased (36.1% vs. 11.4%; *P* = 0.006) [31]. There were also significant differences in urinary indicators. The maximum urine flow rate in the stromal group was significantly lower than in the nonstromal group (7.3 vs. 9.8mL/s; *P* = 0.004) [31]. It was found that as prostate stromal inflammation increased, prostate volume also increased, and symptoms worsened. We know that there are differences in physiological function and in the ratio of stroma to epithelium in the prostate. Inflammation in the stroma and epithelium may affect the progression of BPH and LUTS; stromal inflammation can have an especially profound effect. Distinguishing whether inflammation mainly occurs in prostate stromal tissue or epithelial tissue can improve understanding about the role of inflammation in BPH and LUTS. This finding may also guide clinical treatment. Early treatment for patients with stromal inflammation, such as surgical treatment, may be effective in preventing further enlargement of the prostate and further deterioration of lower urinary tract symptoms, which may improve quality of life.

BPH-induced lower urinary tract symptoms are the main reason patients seek medical treatment. However, the effects of LUTS can be caused by a number of reasons, including systemic inflammation, prostate volume, and the extent to which the prostate protrudes from the bladder. Through observation of patients with obesity or metabolic syndrome, it has been found that lower urinary tract storage symptoms are related to bladder function and prostate inflammation and can be independent to prostate volume [32]. St Sauver et al. [33] analyzed the medical histories of 2,447 Caucasians in Olmsted County and found that the incidence of LUTS was lower in patients with long-term use of nonsteroidal anti-inflammatory drugs (NSAIDs). However, Sutcliffe et al. [34] observed 4,471 BPH patients and used 6 different clinical criteria to conclude that taking NSAIDs was not associated with BPH or LUTS. Interestingly, analysis based on the Finnish National Drug Reimbursement Database shows that patients using NSAIDs have a higher risk of BPH [35]. The observations of Torkko et al. [36] also support this view. They analyzed the clinical data and initial pathological specimens of 859 people from the MTOPS experiment and found that patients taking NSAIDs had a higher risk of BPH and LUTS progression. The relationship between systemic inflammation and BPH/LUTS is still unclear. Some researchers believe that systemic inflammation may cause atherosclerosis in the bladder and prostate blood vessels, leading to LUTS. Whether inflammation affects BPH and LUTS through systemic inflammation or through the prostate itself requires further research.

In short, our understanding of the relationship between inflammation and BPH is only the tip of the iceberg. In addition to cell proliferation, inflammatory cytokines can also affect the phenotype of prostate cells. The difference in the ratio of stromal to epithelial cells in the prostate transition zone, prostate nodule type, and the main inflammatory infiltrated tissue can also lead to differences in LUTS. The direct mechanism by which inflammatory infiltration in the prostate influences LUTS requires further study.

## 4. The Combined Effect of Androgens and Inflammation

Androgens and inflammation are both important factors leading to pathological changes in prostate tissue. They trigger the proliferation of prostate transition zone cells in BPH patients, which causes tissue structure remodeling, and finally induce LUTS. What is the relationship between androgens and inflammation in the occurrence and development of BPH and LUTS? Is there a mutual promotion or inhibition between them?

Many studies have found that androgens in prostate tissue can inhibit inflammation. Quintar et al. [37] treated rats with androgen deprivation (orchiectomy) and found that the expression of TLR4, CD14, and MyD88 (intrinsic immune-related proteins) in prostate tissue gradually increased with time. Western blotting and immunocytochemical analysis showed that the expressions of antibacterial proteins rBD-1 and SP-D gradually increased. Furthermore, E. coli was inoculated into the ventral side of the rat prostate. Five days later, it was found that the number of bacteria in the prostates of the orchiectomy group was significantly lower than the bacteria levels of the control group [37]. Androgens seem to play an important role in maintaining the balance of prostate immunity. Interestingly, Murtola et al. [38] analyzed the results of the prostate cancer prevention trial (PCPT) and found that compared with the control group, the incidence of inflammation in the prostate was significantly increased in both benign and malignant groups after finasteride administration. The trial indicated that after DHT was downregulated in the prostate, the inhibitory effect on inflammation was weakened, and thus, the inflammation of prostate tissue became more obvious. However, the author also pointed out that there was no significant change in PSA levels consistent with increased inflammation [38]; consequentially, it was unclear whether the upregulated inflammation after finasteride administration affected the prostate of BPH patients.

Just as androgens can affect inflammation, inflammation can also affect androgens. Proinflammatory cytokines and immune cells can influence the androgen pathway in the prostate,and synergize with hormones to enhance proliferative stimulation for prostate cells. In 105 prostatectomy specimens, the hyperplastic prostate samples (created by combining three immunohistochemical markers: CD4, CD8, and CD20 assessments) showed clear correlation between immune-mediated inflammation and both prostate volume and AR expression. The immune-mediated inflammation specimens had larger prostate volumes (62.7mL vs. 49.2mL; *P* < 0.05) and higher AR expression levels (56.1% vs. 28.2%; *P* < 0.05) [39]. In contrast to the results of *in vivo* experiments, Debelec-Butuner et al. [40] found that TNF-*α* downregulated the expression of AR and AR target genes (KLK4, PSA, and NKX3.1) in LNCaP and RWPE-1 cells and also downregulated p53, leading to increased gene heterogeneity in prostate epithelial cells. This change could be reversed by addition of anti-TNF antibodies or androgen supplementation. The difference between *in vivo* and *in vitro* experimental results indicates that the internal relationship between prostate inflammation, AR expression, and prostate volume is still unclear. One possible explanation for this contrast may be that some specific inflammatory factors may influence the prostate cells *in vitro*, but the factors' independent effects may not be the real cause of the disease. The key point is whether there is a causal relationship between inflammation and AR expression, and deeper mechanisms require further exploration.

The prostate is mainly composed of supporting stromal tissues and acinar epithelial tissues. The predominance of prostate tissue differed in different types of prostatic hyperplasia nodules, which affect the LUTS of BPH patients [11]. The effects of androgens and inflammation on different tissues in the prostate may also differ. Leimgruber et al. [18] treated pSMCs with lipopolysaccharide (LPS) and testosterone and found that testosterone can significantly inhibit the activation of TLR4 and NF-*κ*B pathways caused by LPS stimulation, thereby inhibiting the expression of proinflammatory factors such as TNF-*α* and IL-6. However, the author observed a very interesting phenomenon, in which testosterone or LPS alone would stimulate pSMC proliferation, but simultaneous addition of testosterone and LPS inhibited stromal cell proliferation [18]. This suggests that the combination of testosterone and inflammation mutually influence the proliferation of pSMCs *in vitro*. Additionally, this influence is not a simple addition of two independent effects. On the contrary, some researchers have observed that DHT treatment itself shows dose-dependent inhibition on the proliferation of prostate epithelial cell lines BPH-1 and PrEC [41]. Further research illustrated that after DHT treatment downregulated the expressions of cellular proliferation-related proteins c-myc and cyclin D1, the expression of CDK (cyclin-dependent kinase) inhibitor p21 was upregulated, and the cell cycle was limited at the G0/G1 phase [41, 42].

The DHT levels in the prostates of BPH patients were significantly higher than those of circulating blood, and there was extensive inflammation in the tissues of the patients' prostates. The effects of DHT and inflammation in the prostate may be related to DHT concentration and the degree or location of inflammation. Zhao et al. [41] focused on the effects of androgens and inflammatory stimulation on macrophages (a type of inflammatory cells that are very common in hyperplastic prostate tissue), rather than their direct effects on prostate tissue. They simulated prostate surgical wounds on experimental canines. At 1–2 weeks, they found that compared with the testosterone injection group, there were significantly fewer M1 macrophages in the prostates of the finasteride group and the control group. The concentration of TNF-*α* at the wound was also lower. At the same time, when DHT was added to low dose, LPS-treated THP-1 cells (a human-derived immortalized monocyte), the expression of TNF-*α* increased significantly, which was not evident in THP-1-shAR cells [41]. DHT can upregulate the expression of inflammatory factor TNF-*α* by stimulating AR on activated macrophages. When constructing a BPH mouse model using exogenous androgens, it was found that the classic inflammation pathway NF-*κ*B was activated in the prostate tissue of mice injected with testosterone subcutaneously, and the expressions of IL-8, TNF-*α*, and COX-2 were significantly upregulated [16, 17]. The microenvironment in the prostate can be affected after testosterone injection, leading to pathological changes. At the same time, testosterone injection induces the activation of inflammatory pathways in the prostate and increases the secretion of inflammatory cytokines. Some researchers administered ACEI (captopril) to mice with testosterone-induced BPH, and ACEI was able to significantly inhibit prostate cell remodeling. Also, the expression of inflammatory cytokines like IL-8 and TNF-*α* in the ACEI group was significantly lower than in the BPH group [17]. It is reasonable to speculate that the expression of AngII could be upregulated after androgen supplementation activates the RAS system in the prostate, which in turn activates the NF-*κ*B inflammatory pathway and causes increased secretion of inflammatory cytokines. This would stimulate the processes of prostate tissue damage and repair, resulting in BPH development. At the same time, due to the activation of the RAS system in the prostate, blood vessels and ducts would become blocked and calcified, which would further aggravate inflammation infiltration and ultimately lead to LUTS.

Another group of researchers performed a similar experiment. They gave saw palmetto to mice with testosterone-induced BPH and found that compared with BPH mice, prostate volume and weight were significantly reduced, prostatic cell hypertrophy was significantly improved, and the level of inflammatory cytokines was downregulated. The effect was similar to that of the classic BPH treatment drug finasteride. Interestingly, the researchers found that in addition to the antiandrogenic effect, saw palmetto also shows an anti-inflammatory effect, which can downregulate B lymphocyte infiltration in the prostate and reduce the levels of IL-1 and TNF-*α* [43, 44]. At present, most scholars believe that pathogenic infection and lymphocyte aggregation caused by urine reflux are the main causes of prostatic inflammation, but clinically, BPH has not been observed to be associated with prostatitis. Perhaps, the combination of DHT and mild inflammation in the prostate transition zone amplify the expression of inflammatory factors and upregulate the proliferation ability of prostate tissue, instead of inflammatory infiltration as the main manifestation. The DHT levels in the prostates of BPH patients and the predisposing factors of inflammation deserve more in-depth study.

Stromal, epithelial, and immune cells are the main components of hyperplastic prostate tissue. BPH is the result of the combined effects of different factors on the three components. Studying the roles of and relationship between androgens and inflammation in prostate stromal, epithelial, and immune cells will improve understanding of the pathogenesis of BPH and LUTS and enable the creation of more effective treatment plans in the clinical setting.

## 5. Conclusion

Although our understanding of the mechanism by which androgen changes and inflammation induce BPH in the prostate remains incomplete, current research indicates that changes in androgen levels and AR expression in the prostate, as well as chronic inflammation, play important roles in the occurrence and progression of BPH and LUTS. BPH is a comprehensive manifestation of various factors, especially androgens and inflammation, on the stimulation of prostate intraepithelial and stromal tissues. If the influence of androgens and prostate inflammation on BPH progression can be understood, clinicians can be better supported and more suitable treatment plans can be made.
